# The Effect of Chitosan’s Addition to Resorcinol/Formaldehyde Xerogels on the Characteristics of Resultant Activated Carbon

**DOI:** 10.3390/ma12233847

**Published:** 2019-11-22

**Authors:** Ahmed Awadallah-F, Shaheen A. Al-Muhtaseb

**Affiliations:** Department of Chemical Engineering, Qatar University, Doha, P.O. Box 2713, Qatar; ahmed.awadallah@qu.edu.qa

**Keywords:** resorcinol, formaldehyde, xerogel, chitosan, activated carbon

## Abstract

Hybrid chitosan-resorcinol/formaldehyde xerogels were synthesized, and the effect of including minor quantities of chitosan on the consequent activated carbon was investigated. The resulting activated carbon were characterized by different techniques. Clear changes were found in the structure of activated carbon as a result of including chitosan in the synthesis. The results showed that the disorder ratio of crystal lattice decreased from 0.750 to 0.628 when increasing the concentration of chitosan from 0 to 0.037 wt%. The micropores increased from ~0.3% to ~1.0%, mesopores increased from ~11.2% to ~32.9% and macropores decreased from ~88.4% to ~66.1%. The total pore volume decreased from 1.040 to 0.238 cm^3^/g and the total pore surface area decreased from 912.3 to 554.4 m^2^/g. On the other hand, the average pore width decreased from 2.3 to 0.8 nm and the average particle size decreased from 224 to 149 nm. Nano-scale Scanning Electron Microscope (NanoSEM) morphology indicated a critical composition of chitosan (0.022 wt%) that affects the structure and thermal stability of activated carbon produced.

## 1. Introduction

Resorcinol/formaldehyde xerogels can be converted to activated carbon (Resorcinol/formaldehyde xerogel activated carbon, RFX-AC) that has hierarchical pore structures ranging from micropores to macropores [1,2,3], which gives it an advantage over single-sized porous materials [4]. On one hand, the large macropores improve permeability and ion diffusion; and on the other hand, the mesopores create low resistance pathways through the material matrix. Furthermore, the smaller pores (either micropores or mesopores) increase the specific surface area and the pore volume. This enhances the adsorption capacity of the carbon and provides high capacitance for super-capacitors [5,6,7]. There are various methods with which to prepare activated carbon xerogels, such as the carbonization and activation of organic gels [8,9]; derivation from biomass [10]; and the hydrothermal reduction of graphene oxide [11]. Among these methods, the carbonization and activation of organic gels is the most widely used [12]. Many efforts have been exerted to tailor and design the pore structures of activated carbon gels by changing their synthesis conditions or by adding agents (additives) [13]. Such additives are very important to enhancing the properties of activated carbon in order to widen its application range and increase the efficiency of the resulting carbons as well. One of these additives is chitosan (Cs), which is a natural random linear copolymer of β-(1–4)-linked D-glucosamine and N-acetyl-D-glucosamine [14]. Cs is utilized as a biosource to get activated carbon for numerous applications, such as H_2_ and CO_2_ storage [15,16], electrodes for super-capacitors [17] and batteries [18]. The fundamental factors which make Cs important are its availability as a byproduct of the food industry and its high carbonization yield (~50 wt%), which improves the electronic structure and wettability of material’s surface as well [19]. Therefore, the addition or insertion of heteroatoms, such as oxygen, nitrogen, fluorine or phosphorus (of which some are involved in the Cs structure) enhances the capacity of carbon electrode by inducing pseudocapacitance [20,21,22].

The aim of this work was to study the effect of Cs addition, in minor quantities during the synthesis of hybrid resorcinol/formaldehyde xerogels [23], on the resultant activated carbon. Various techniques were used to characterize the activated carbon that is produced, including Fourier transform infrared (FTIR), Raman spectrometry, thermal gravimetric analysis (TGA), X-rays diffraction (XRD), NanoSEM, N_2_ gas adsorption/desorption isotherms and porosimetry (pore size distributions as pore surface areas and volumes) through the density functional theory (DFT) model.

## 2. Materials and Methods

### 2.1. Materials

Resorcinol (purity 99%) and formaldehyde solution (37 wt% in H_2_O with 10%–15% methanol as the stabilizer) were purchased from Sigma-Aldrich, Germany. Sodium carbonate (Na_2_CO_3_) anhydrous catalyst was purchased from Fisher Scientific, Loughborough,, UK. Ultra-pure water was supplied from a Millipore Elix^®^70 Water Purification System, Molsheim, France. Chitosan (medium molecular weight) and deacetylated chitin were supplied by Sigma-Aldrich, St. Louis, MO, USA. Other reagents (acetone, acetic acid, nitric acid and ammonium hydroxide) were of analytical reagent grade. All chemicals were used as received.

### 2.2. Carbonization and Activation Processes

Six hybrid, xerogel, activated carbon samples (RFX-Cs-AC-0 through RFX-Cs-AC-5) were prepared, for which the suffix number reflects to the amount of Cs used in the precursor gel. Full details of gels synthesis are found elsewhere [23]; and the suffix numbers 0 through 5 correspond, respectively, to the Cs concentration in the starting solutions ranging from 0 to 0.037 wt%, as listed in Table 1 [23]. The use of such low concentrations of Cs was due to the very little solubility of Cs into the initial solution media. Each dried RFX-Cs sample was placed in a ceramic crucible inside a programmable electric-heated tube furnace (Nabertherm GmbH, Bremen, Germany), with a continuous flow (100 cm^3^/min) of N_2_ gas. The furnace was first kept at ambient temperature for 30 min (while flowing N_2_) to make sure that air is entirely purged out. Then, the sample was heated up to a temperature of 500 °C with a heating rate of 10 °C/min; kept at 500 °C for 3 h; and then let to cool off spontaneously to ambient temperature while N_2_ gas was flowing. Afterwards, the carbonized samples were activated in the same tube furnace (after cleaning it comprehensively from the residues of the carbonization process) with carbon dioxide gas flow (150 cm^3^/min) instead of N_2_ gas; heating of the sample again with a rate of 10 °C/min to 700 °C; keeping it at this temperature for 1 h; and then letting the sample to cool down spontaneously to ambient temperature while flowing CO_2_ gas [24]. After carbonization and activation processes of each RFX-CS-n (where n corresponds to the designated sample number), each corresponding activated carbon sample was called RFX-CS-AC-n, where n is the same number as the precursor RFX-CS-n [23] and corresponds to the concentration of Cs in the starting solution (Table 1).

### 2.3. Characterization

Fourier transform infrared (FTIR) spectroscopy (NICOLET, iS10, Thermo-Scentific, Waltham, MA, USA) was used to confirm the structure of the samples. FT-Raman spectra were estimated by a Bruker FT-Raman spectrometer of type RFS 100/S (Bruker, New York, NY, USA ) that was attached to a Bruker-IFS 66/S spectrometer (Thermo Fisher Scinetfiic, Madison, WI, USA) with a high resolution to better than 0.10 cm^−1^. The morphologies of RFX-CS-AC samples were observed with a FEI Nova™ nanoscanning electron microscopy 450 (Nova NanoSEM, Prague, Czech Republic); and the elemtal analysis was conducted by a SEM-attached Energy-dispersive X-ray (EDX). Thermogravimetric analyses (TGA) were carried out in the range of 30 to 800 °C (with a heating rate of 10 °C/min) using a PerkinElmer Pyris6 TGA analyzer (Perkinelmer, Waltham, MA, USA) under a flow of N_2_ gas. X-ray diffraction (XRD) measurements were conducted by Miniflex-II Benchtop XRD apparatus, manufactured by Rigaku Corporation, Tokyo, Japan. The 2θ scan data were collected over the range of 5° to 90° at 0.05° intervals and at a scan rate of 0.05°/min. A Micromeritics ASAP2420^®^ accelerated surface area and porosimetry analyzer (Micromeritics, Norcross, GA, USA), with an enhanced micropore capability (utilizing 1-Torr pressure transducer, Micromeritics, Norcross, GA, USA), was used to measure the pore structures of RFX-CS-AC samples using the adsorption/desorption isotherms of N_2_ at 77 K. Prior to the adsorption/desorption isotherms measurements, the samples were regenerated in situ for 24 h at a temperature of 473 K under vacuum (1 × 10^−4^ Pa). The pore properties data were determined by built-in calculations based on the density functional theory (DFT).

## 3. Results and Discussion

**Figure 1:**
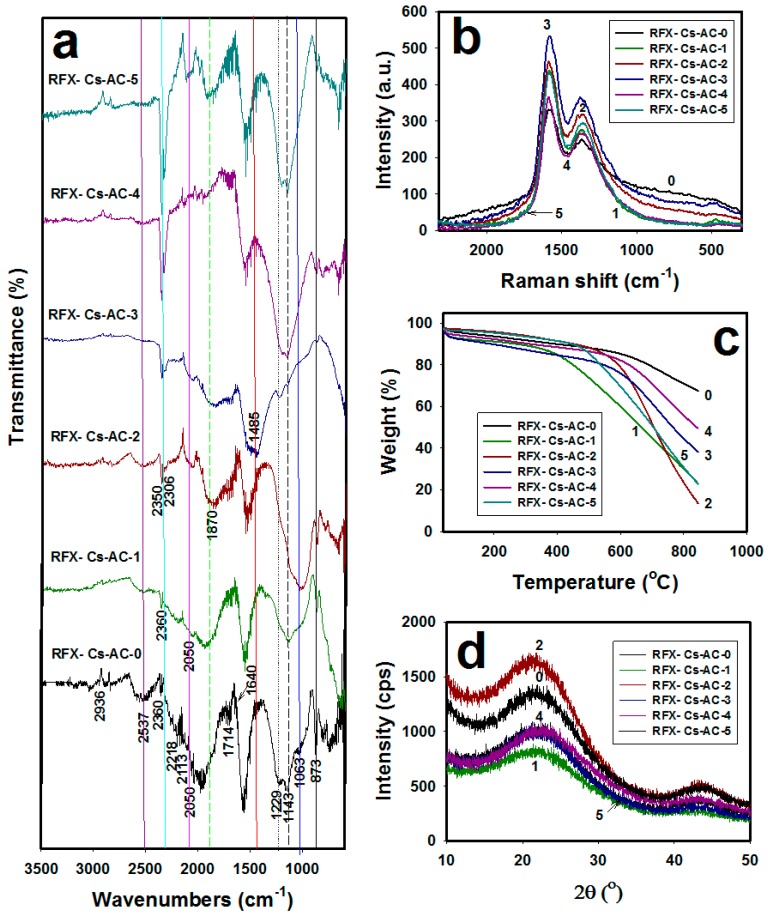
(**a**) FTIR spectra, (**b**) Raman spectra, (**c**) TGA thermograms and (**d**) XRD patterns of RFX-Cs-AC samples. Curve numbers from 0 to 5 correspond to the suffix numbers of RFX-Cs-Ac samples.

Figure 1a shows the FTIR spectra of the six RFX-Cs-AC samples. The peak at 2936 cm^−1^ was attributed to stretching of –CH_2_ groups. The peaks observed at 1063, 1143 and 1229 cm^−1^ are related to the C–O–C linkage stretching between two resorcinol molecules, which is assignable to the polycondensation between resorcinol and formaldehyde molecules. The peak at 1640 cm^−1^ can be attributed to aromatic stretching. Peaks observed at 2360 and 2218 cm^−1^ can be attributed to C≡C, which results from breaking benzene rings with heating to form –C≡C– bonds at the broken ends [25]. The peak at 1714 cm^−1^ can be seen clearly in the RFX-Cs-0 and it can be attributed to C=O group. This may be either from carboxylic (COOH) or quinone groups, which may be formed at the broken C–O–C linkages. The peaks at 873 cm^−1^ could be due to C–C vibrations. The peak at 2050 cm^−1^ is assignable to the carbonyl group from the quinone group [26,27]. The peaks at 2113 cm^−1^ was attributed to the stretching of carbonyl group. It was observed that the intensity of peak at 873 cm^−1^ (black line) decreased from RFX-Cs-AC-1 to RFX-Cs-AC-5 by increasing Cs into the matrix of RFX-AC. The peak at 1073 cm^−1^ (blue line) appeared in RFX-Cs-AC-0, RFX-Cs-AC-1 and RFX-Cs-AC-2 and disappeared in RFX-Cs-AC-3, RFX-Cs-AC-4 and RFX-Cs-AC-5. The peaks at 1143 and 1229 cm^−1^ (dashed black line and dotted black line) appeared in all samples as big bands except for RFX-Cs-AC-3, where it appeared weakly. The peak at 1485 cm^−1^ (red line) appeared only as broad band for RFX-Cs-AC-3 and disappeared from others. A broad peak at 1870 cm^−1^ (dashed green line) appeared from RFX-Cs-AC-1 to RFX-Cs-AC-5. The peak at 2050 cm^−1^ (pink line) appeared clearly in samples of RFX-Cs-AC-1 to RFX-Cs-AC-5, and weakly in RFX-Cs-AC-0. The intensity and sharpness of the peak at 2360 cm^−1^ (cyan line) increased by increasing Cs. The broad peak at 2537 cm^−1^ (dark pink line) decreased by increasing Cs.

Figure 1b exhibits the Raman spectra of the RFX-Cs-AC samples. It was seen that there were two characteristic peaks at 1570 cm^−1^ and 1360 cm^−1^. The first peak (G-band) refers to graphitic structure, and its intensity (indicated as *I_G_*) represents the order of the crystal lattice of each material. The second peak (D-band) represents the disorder/defects of crystal lattice, and its intensity is denoted as *I_D_* [28]. The ratio of *I_D_*/*I_G_* estimates the relative disorder of materials. Overall, through the results of *I_D_/I_G_* in Table 1, it can be seen that increase of Cs into the matrix of RFX-AC occurs to the decrement of *I_D_/I_G_*, consequently increasing the defects/disorders into the structure.

**Figure 2:**
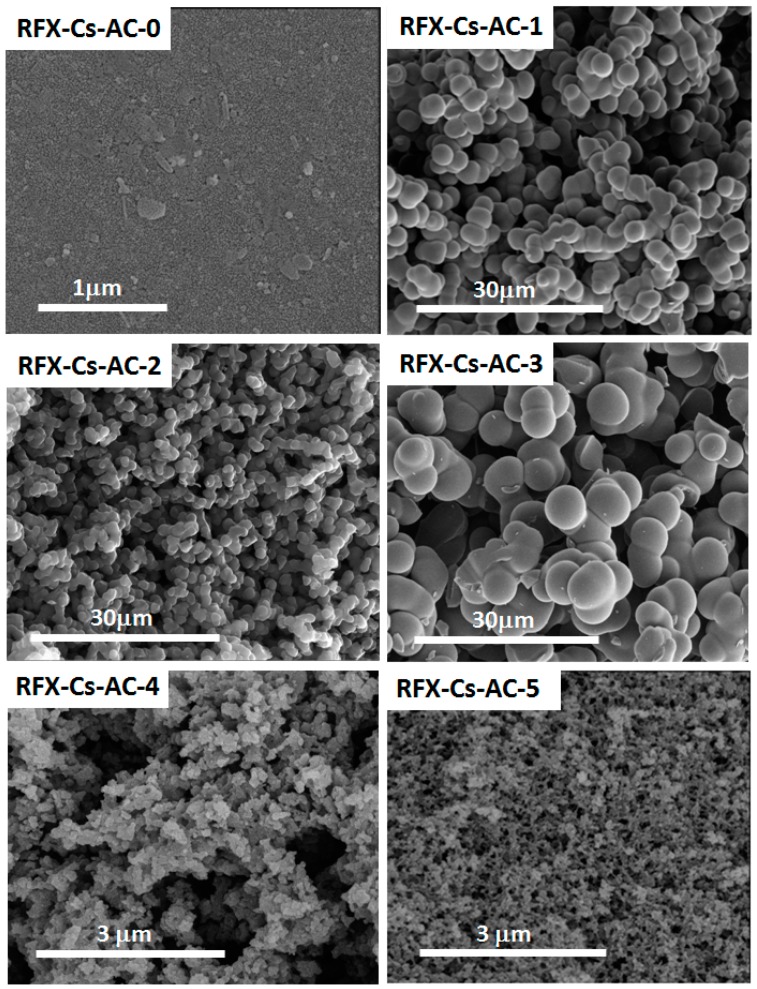
NanoSEM photomicrographs of RFX-Cs-AC samples.

Figure 1c shows the TGA thermograms of RFX-Cs-AC samples, where it was observed that the addition of Cs into the matrix of RFX-AC leds to noticeable changes in its thermal stability. The weight losses of RFX-Cs-AC samples at different temperatures are listed in Table 2. Overall, the order of thermal stability up to ~400 °C (according to RFX-Cs-AC sample numbers) was 2 ≈ 5 > 0 > 4 > 1 > 3. Then, up to ~470 °C, the order became 2 ≈ 5 > 0 > 4 > 3 > 1. Then, up to ~490 °C, the order became 2 ≈ 0 > 5 > 4 > 3 > 1. Afterwards, up to ~530 °C, the order became2 ≈ 0 > 4 > 5 > 3 > 1. Then, up to ~570 °C, the order became 0 > 2 ≈ 4 > 5 > 3 > 1. Then, up to 700 °C the order became 1 > 5 >2 >3 >4 >0. At 800 °C, the order became 2 > 1 ≈5 > 3 > 4 > 0. Finally, at 845 °C, the order became 2 > 1≈5 >3 > 4>0. Therefore, the addition of Cs in minor concentrations can have a significant effect on the thermal stability. Figure 1d illustrates the XRD patterns of RFX-Cs-AC samples. Broad peaks at 2θ of 21° and 43° appeared for all the samples with different intensities, which indicates that the structures of RFX-Cs-AC samples are amorphous [29]. From the diffractograms, it can be seen that RFX-Cs-AC-2 was the most amorphic, while RFX-Cs-AC-1 was the least among the samples. Figure 2 exposes the photomicrographs of RFX-Cs-AC samples. As an overview, it can be said that the addition of Cs onto the matrix of RFX-AC leads to a change into the morphology of sample produced. Further, the samples of RFX-Cs-AC-3 may be considered to resemble a critical composition among samples, after which the sample shapes change from big spheres to small spheres. This composition corresponds to 0.022 wt% of Cs in the starting solution [23].

**Figure 3:**
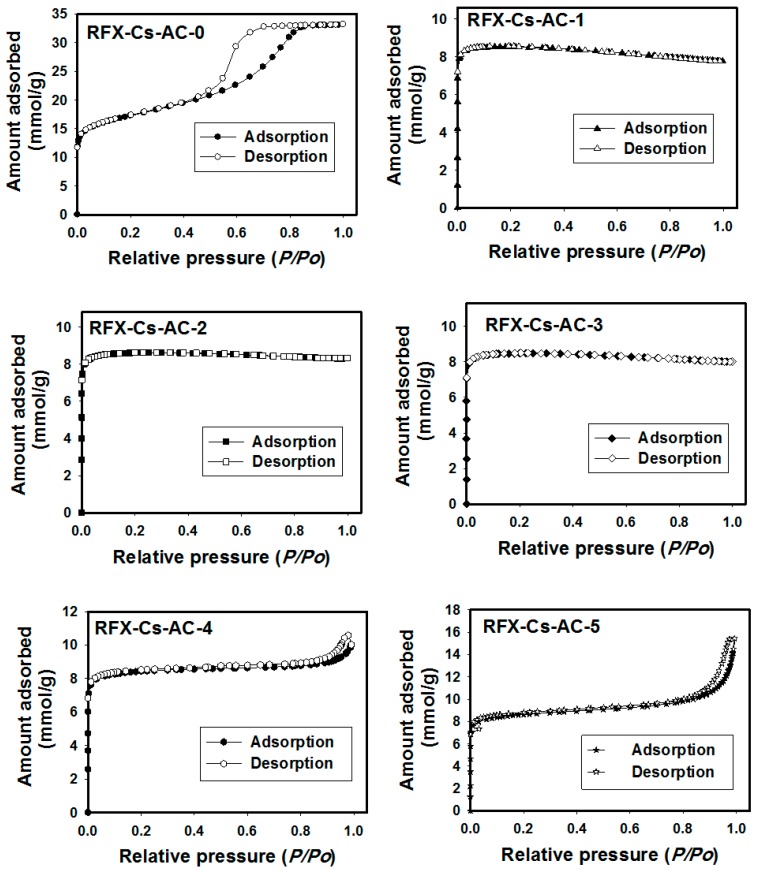
Adsorption/desorption isotherms of N_2_ gas at 77 K onto RFX-Cs-AC samples.

Figure 3 shows the N_2_ adsorption/desorption isotherms of RFX-Cs-AC samples. It is apparent from subgraphs that the shape of isotherm on RFX-Cs-AC-0 is of type IV. Among the characteristic features of a Type IV isotherm are its hysteresis loop, which is associated with capillary condensation taking place in mesopores, and the limiting uptake over a range of high *P*/*P_0_* values [30]. The hysteresis loop is usually attributed to the thermodynamic or network effects or the combination of these two effects. This hysteresis loop refers to pores with narrow mouths (ink-bottle pores); relatively uniform, channel-like pores; and pore network (connectivity) effects [31].

Generally, it was noticed from Table 1 that N_2_ adsorption capacity decreased by increasing Cs addition into the matrix of the RFX-Cs precursor, but it started to increase again after RFX-Cs-AC-3. The micropores, mesopores and macropore percentages were almost constant from RFX-Cs-AC-0 to RFX-Cs-AC-3, and then the percentages of micropores and mesopores increased while those of macropores decreased by adding Cs. Further, the pore width decreases by increasing Cs addition into the matrix of RFX-AC from 2.3 nm (for RFX-Cs-AC-0) to 0.8 nm (for RFX-Cs-AC-1 to RFX-Cs-AC-3); then, it increases again for RFX-Cs-AC-4 and RFX-Cs-AC-5. It can be deduced that RFX-Cs-AC-0 exhibits the highest adsorption capacity among samples. This is in agreement with the values of N_2_ adsorption capacity and pore widths reported in Table 1. The same conclusion can be noticed from Table 1 about the total pore volume decreasing with increasing Cs addition from 1.0 cm^3^/g (for RFX-Cs-AC-0) to 0.2 cm^3^/g (for RFX-Cs-AC-1 to RFX-Cs-AC-3); then, it increases again for RFX-Cs-AC-4 and RFX-Cs-AC-5. The total surface area decreases by increasing Cs addition from 912 m^2^/g (for RFX-Cs-AC-0) to 554 m^2^/g (for RFX-Cs-AC-1). Then, it increases slightly with the addition of Cs. Furthermore, it is apparent from particle size results in Table 1 that increasing of Cs into the matrix of RFX-Cs increases the particle size to reach the maximum at 795.2 nm of RFX-CS-AC-3, and then decreases to reach 148.8 nm of RFX-CS-AC-5. Therefore, RFX-CS-AC-3 can be considered to be a critical composition among samples. This composition corresponds to 0.022 wt% of Cs in the starting solution [23]. Moreover, Table 1 indicates that the percentage of activated carbon (AC%) which corresponds to the yield of activated carbon produced from the precursor gels, decreases with more Cs in the starting solution of the precursor gel.

**Figure 4:**
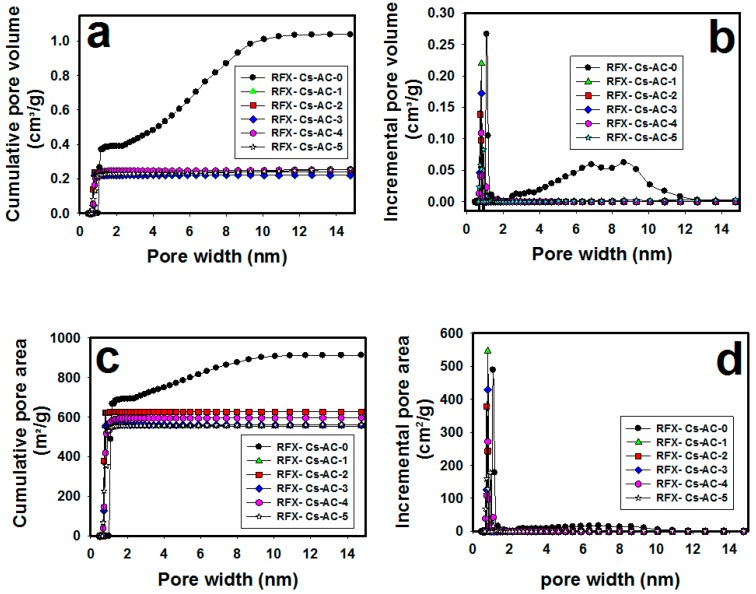
Relationships of (**a**) cumulative pore volume, (**b**) incremental pore volume, (**c**) cumulative pore area and (**d**) incremental pore area versus pore widths of different RFX-Cs-AC samples.

Figure 4a–d shows the relationships between pore width and cumulative pore volume, incremental pore volume, cumulative pore area and the incremental pore area of each RFX-Cs-AC sample, respectively. It was seen from Figure 4a that the cumulative pore volume of RFX-Cs-AC-0 began to increase from pore width 0.5 to 10 nm, whereas the pore volumes of other samples (of RFX-Cs-AC-1 to RFX-Cs-AC-5) were constantly above about >0.5 nm. Further, the cumulative pore volume of RFX-Cs-AC-0 (1.037 cm^3^/g) represents the highest value, whereas that of RFX-Cs-AC-3 (0.223 cm^3^/g) represents the lowest value. Figure 4b exposes the distribution of incremental pore volume against pore width. The incremental pore volume of RFX-Cs-AC-0 represents the highest value—0.266 cm^3^/g at 1.1 nm. The maximum incremental pore volumes of RFX-Cs-AC-1, RFX-Cs-AC-2, RFX-Cs-AC-3, RFX-Cs-AC-4 and RFX-Cs-AC-5 were, respectively, 0.219, 0.138, 0.171, 0.108 and 0.058 cm^3^/g at the corresponding pore width of 0.8 nm. There are two peaks of RFX-Cs-AC-0 at 6.8 and 8.6 nm with the corresponding incremental pore volumes of 0.058 and 0.0621 cm^3^/g, respectively. Further, no significant incremental pore volumes were observed for the samples RFX-Cs-AC-1 to RFX-Cs-AC-5 at >1 nm. It was also noticed from Figure 4c that the cumulative pore area of RFX-Cs-AC-0 increases by increasing pore width up to 992 m^2^/g at a pore width of 9.3 nm. No increase was noticed in the cumulative pore area of RFX-Cs-AC-2 at pore width >0.8 nm, which reached a plateau at 629 m^2^/g. Meanwhile the cumulative pore areas of RFX-Cs-AC-1, RFX-Cs-AC-3, RFX-Cs-AC-4 and RFX-Cs-AC-5 leveled off at a pore width of ~1.4 nm. The maximum values of cumulative pore areas of RFX-Cs-AC-1, RFX-Cs-AC-3 and RFX-Cs-AC-5 were almost identical at ~561.316 m^2^/g, whereas that of RFX-Cs-AC-4 was 597 m^2^/g. Figure 4d illustrates the distribution of the incremental pore areas versus pore width. It was seen that RFX-Cs-AC-1 represented the highest incremental pore area among samples (546 m^2^/g) at 0.8 nm, whereas RFX-Cs-AC-5 represented the lowest value (178 m^2^/g) at 1.1 nm. Further, the sequential order of the maximum incremental pore areas regardless of the corresponding pore widths was RFX-Cs-AC-1 > RFX-Cs-AC-0 > RFX-Cs-AC-3 > RFX-Cs-AC-2 > RFX-Cs-AC-4 > RFX-Cs-AC-5. In conclusion, as a general deduction of aforementioned results, it can be said that the addition of Cs into the matrix of RFX affects, significantly, the composition and character of the RFX-Cs-AC samples.

## 4. Conclusions

This study tackled the synthesis of activated carbon xerogels from gelled composites of resorcinol/formaldehyde with chitosan as a minor additive. The addition of chitosan in minor concentrations (up to 0.037 wt%) to the precursor gel composites led to noticeable changes in the properties of the consequent activated carbon. These properties were characterized by FTIR, Raman spectra, TGA, XRD, NanoSEM, EDX, N_2_ adsorption/desorption isotherms and surface area and porosimetry analyses. The results showed that the chemical structure, crystal order, thermal stability, morphology and pore structures of these activated carbon were clearly affected by the presence of chitosan as a minor additive. The minor concentrations of chitosan that were added during the synthesis of precursor gels that were then converted into activated carbon had significant impacts on the properties of the activated carbon batches produced. The results indicate that the disorder ratio of crystal lattice decreased from 0.750 to 0.628 by increasing the concentration of Cs from 0 to 0.037 wt%. The micropores increased from ~0.3 to ~1.0%, mesopores increased from ~11.2 to ~32.9% and macropores decreased from ~88.4 to ~66.1%. The total pore volume decreased from 1.040 to 0.238 cm^3^/g and total pore surface area decreased from 912.3 to 554.4 m^2^/g. Moreover, the average pore width decreased from 2.3 to 0.8 nm and the average particle size decreased from 224 to 149 nm. Future studies of these hybrid carbons will focus on more variable parameters in the synthesis process and on different applications, such as their use for the adsorption of gases (e.g., CO_2_ gas capture) to evaluate the effect of Cs as a minor addition on activated carbon’s behaviors.

## Figures and Tables

**Table 1:** Structural characteristics of resorcinol/formaldehyde xerogel activated carbon with chitosan (RFX-Cs-AC) samples.

Sample	Cs in Starting Solution (wt%)	V_Total_^a^ (cm^3^/g)	S_Total_^a^ at pore ≥ 0.5 nm (m^2^/g)	Particle Size ^b^ (nm)	N_2_ Adsorption Capacity ^a^ (mmol/g)	Pore Width ^c^ (nm)	Micro-pores ^c^ (%)	Meso-pores ^c^ (%)	Macro-pores ^c^ (%)	EDXanalysis (wt%)		(*I_D_/I_G_*) ^d^	AC (%)
										C	O		
RFX-Cs-AC-0	0	1.0 at pore ≤ 400.0 nm	912	224	33.2	2.3	0.3	11.2	88.5	98.8	1.2	0.750	38.3
RFX-Cs-AC-1	0.007%	0.2 at pore ≤ 370.7 nm	554	280	7.8	0.8	0.4	12.1	87.5	98.1	1.9	0.628	25.1
RFX-Cs-AC-2	0.015%	~0.2 at pore ≤ 400.3 nm	627	403	8.3	0.8	0.3	11.3	88.4	98.9	1.1	0.696	22.2
RFX-Cs-AC-3	0.022%	0.2 at pore ≤ 400.3 nm	561	795	8.6	0.8	0.3	11.3	88.4	98.0	2.0	0.683	20.0
RFX-Cs-AC-4	0.029%	0.3 at pore ≤ 136.7 nm	598	184	10.6	1.0	1.0	32.9	66.1	98.5	1.5	0.718	21.1
RFX-Cs-AC-5	0.037%	0.4 at pore ≤ 185.8 nm	592	149	15.4	1.4	0.8	24.1	75.1	99.0	1.0	0.682	18.0

^a^ Total pore volume (V_Total_) and total surface area (S_Total_) were determined from density functional theory (DFT) and N_2_ adsorption/desorption isotherms at 77 K (via the Micromeritics ASAP2420^®^ analyzer, Micromeritics, Norcross, GA, USA). ^b^ Average particle size distribution obtained from the Micromeritics ASAP2420^®^ analyzer. ^c^ Refers to the average pore sizes of micro-, meso- and macropores based on the incremental surface area values of DFT analysis. ^d^ Extracted from Raman spectra analysis.

**Table 2:** TGA weight losses (%) of RFX-Cs-AC samples.

Sample	Temperature (°C)								
	100	200	300	400	500	600	700	800	845
RFX-Cs-AC-0	4.6	6.3	8.2	10.0	11.9	14.7	20.9	29.1	32.5
RFX-Cs-AC-1	7.4	8.8	10.6	15.2	26.2	40.1	55.1	70.0	77.2
RFX-Cs-AC-2	3.0	4.0	6.0	8.5	11.8	21.2	48.1	76.5	86.5
RFX-Cs-AC-3	7.9	10.2	12.8	15.3	17.9	24.1	38.5	55.4	61.8
RFX-Cs-AC-4	6.0	7.6	9.7	11.5	13.5	17.8	29.1	44.3	50.5
RFX-Cs-AC-5	3.6	4.6	6.4	8.5	14.4	30.4	49.2	69.2	77.1
